# What Has Been Trending in the Research of Polyhydroxyalkanoates? A Systematic Review

**DOI:** 10.3389/fbioe.2020.00959

**Published:** 2020-09-10

**Authors:** Maciej Guzik, Tomasz Witko, Alexander Steinbüchel, Magdalena Wojnarowska, Mariusz Sołtysik, Sławomir Wawak

**Affiliations:** ^1^Jerzy Haber Institute of Catalysis and Surface Chemistry Polish Academy of Sciences, Kraków, Poland; ^2^Institut für Molekulare Mikrobiologie und Biotechnologie, Westfälische Wilhelms-Universität Münster, Münster, Germany; ^3^Environmental Sciences Department, King Abdulaziz University, Jeddah, Saudi Arabia; ^4^Department of Product Technology and Ecology, Cracow University of Economics, Kraków, Poland; ^5^Department of Management Process, Cracow University of Economics, Kraków, Poland

**Keywords:** polyhydroxyalkanoate, data mining, trends, composites, medicine, renewable production, polyhydroxyalkanoate (PHA) synthesis, biotechnology

## Abstract

Over the past decades, enormous progress has been achieved with regard to research on environmentally friendly polymers. One of the most prominent families of such biopolymers are bacterially synthesized polyhydroxyalkanoates (PHAs) that have been known since the 1920s. However, only as recent as the 1990s have extensive studies sprung out exponentially in this matter. Since then, different areas of exploration of these intriguing materials have been uncovered. However, no systematic review of undertaken efforts has been conducted so far. Therefore, we have performed an unbiased search of up-to-date literature to reveal trending topics in the research of PHAs over the past three decades by data mining of 2,227 publications. This allowed us to identify eight past and current trends in this area. Our study provides a comprehensive review of these trends and speculates where PHA research is heading.

## Introduction

Polyhydroxyalkanoates (PHAs) have been researched since their discovery in 1920. An exponential burst of scientific publications started in the early 1990s. Since then, yearly, more and more scientific documents are appearing, increasing our knowledge in this realm of biopolymers. A systematic literature review is an important method of understanding the field of study. However, due to high labor intensity, it is usually limited to narrow subjects. They are, in most cases, narrative and qualitative (Tranfield et al., 2003). To study the whole field, it is necessary to adopt text-mining toolset and quantitative methods. It is relatively easy to perform analysis based on metadata related to publications, mostly titles, keywords, and abstracts. The required software is readily available. Unfortunately, research by Blake (2010) revealed that authors report <8% of scientific claims in abstracts. Moreover, keywords used in publications are in many cases limited or modified by publishers. Therefore, studies based on metadata cannot be treated as fully reliable. The following limitation of most text-mining studies is predefinition of themes, clusters, or categories. This can prevent the discovery of new topics that were not predefined by researchers. Additionally, in the case of qualitative methods, the risk of researcher bias grows significantly.

**Figure 1:**
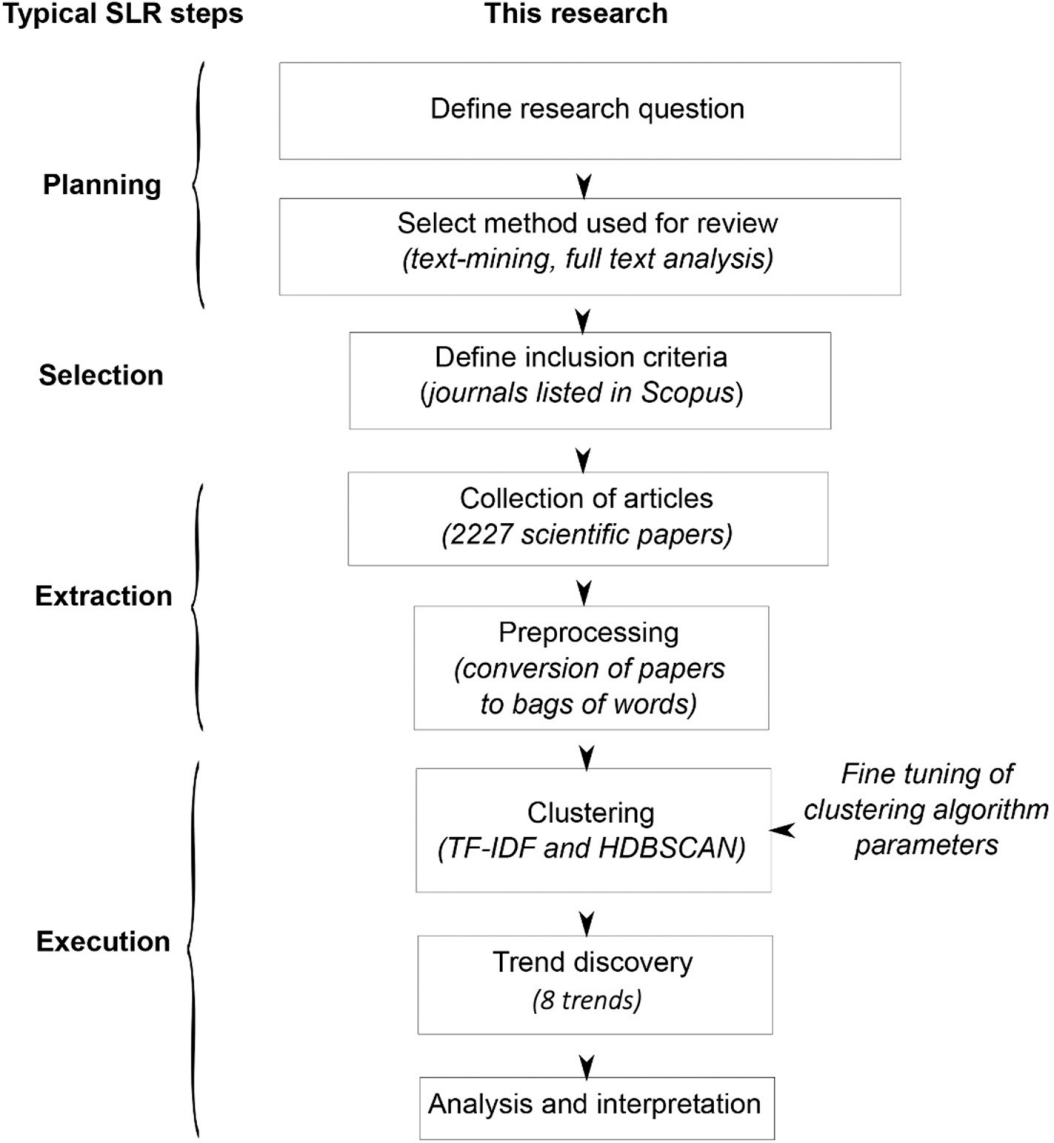
Diagram of the method and steps of systematic literature review (SLR). HDBSCAN, Hierarchical Density-Based Spatial Clustering of Applications with Noise; TF-IDF, term frequency–inverted document frequency.

The approach offered in this study was designed to overcome such limitations. The analysis was performed using full texts of publications, and categories were discovered in data, not predefined. The well-known quantitative approach to text-mining was supplemented with new and original tools (for a full description, please see the Materials and Methods section). This allowed authors to identify not only categories but also trends. A diagram of the proposed approach compared with systematic literature review steps is presented in Figure 1. The procedure is consistent with general rules for systematic literature reviews (Ananiadou et al., 2009). In this article, we have sampled scientific publications from Web of Science Core Collection that were firmly related to PHA research and performed an *in silico* analysis of the most frequently appearing words within the main text. We have omitted on purpose reviews that are not original *per se*, thus focusing only on scientific publications concerning the generation of new data in areas related to PHAs at the time of their publications. This allowed us to identify scientifically relevant keywords that enabled us to group the publications into clusters, groups, and then trends that have been emerging since the late 1980s.

## Finding Trends

### Collection of Publications

In order to collect publications, it was necessary to define search criteria, which included:

- the main subject of a publication related to PHA polymers (search topic term “polyhydroxyalkanoate”),
- availability of the full text of the publication,
- published between 1988 and 2018,
- only scientific publications were included.

The search was performed in the scientific database Web of Science Core Collection using the command “all fields.” In the result, 2,432 publications were found. Publications from 1988 to 1994 were excluded from the analysis due to an overall small number over these years (34 publications). After removing duplicated, editorials, reviews, and other non-scientific publications, 2,227 studies were left. The number of publications ordered by journal title was presented in Table 1. Only publications from journals, which published more than 10 studies in total with relation to the keyword “polyhydroxyalkanoate” were presented in this table.

**Table 1:** Number of publications in polyhydroxyalkanoate (PHA) research by journal.

Applied Microbiology and Biotechnology	139
Applied and Environmental Microbiology	108
Bioresource Technology	86
International Journal of Biological Macromolecules	69
Fems Microbiology Letters	68
Biomacromolecules	60
Journal of Biotechnology	57
Journal of Bioscience and Bioengineering	54
Polymer Degradation and Stability	46
Biotechnology and Bioengineering	36
Biotechnology Letters	35
New Biotechnology	34
Journal of Bacteriology	28
Journal of Polymers and the Environment	28
Water Research	28
Applied Biochemistry and Biotechnology	25
Journal of Applied Microbiology	25
Journal of Chemical Technology and Biotechnology	25
Water Science and Technology	25
Microbial Cell Factories	23
Journal of Industrial Microbiology Biotechnology	21
Macromolecular Bioscience	21
Canadian Journal of Microbiology	20
Polymers	19
Process Biochemistry	19
World Journal of Microbiology Biotechnology	19
Environmental Microbiology	18
Microbial Biotechnology	18
Plos One	18
Biomaterials	17
Journal of Applied Polymer Science	17
Biochemical Engineering Journal	16
Metabolic Engineering	16
Abstracts of Papers of the American Chemical Society	15
Frontiers in Microbiology	15
AMB Express	14
Bioscience Biotechnology and Biochemistry	14
European polymer Journal	14
Journal of Microbiology and Biotechnology	14
Microbiology Resource Announcements	14
Microbiology Sgm	14
Biotechnology Journal	13
Applied Food Biotechnology	12
Indian Journal of Microbiology	12
International Journal of Systematic and Evolutionary Microbiology	12
Biotechnology and Bioprocess Engineering	11
ACS Symposium Series	10
Antonie Van Leeuwenhoek International Journal of General and Molecular Microbiology	10
Bioprocess and Biosystems Engineering	10
Current Microbiology	10
Enzyme and Microbial Technology	10
Other Journals	765

Each publication was converted into a text file and prepared for automatic analysis using computer algorithms. In this research, algorithms have been created using Python libraries, including grobid, nltk, scikit-learn, hdbscan, and scipy (Jones et al., 2001; Lopez, 2009; Pedregosa et al., 2011; McInnes et al., 2017).

**Figure 2:**
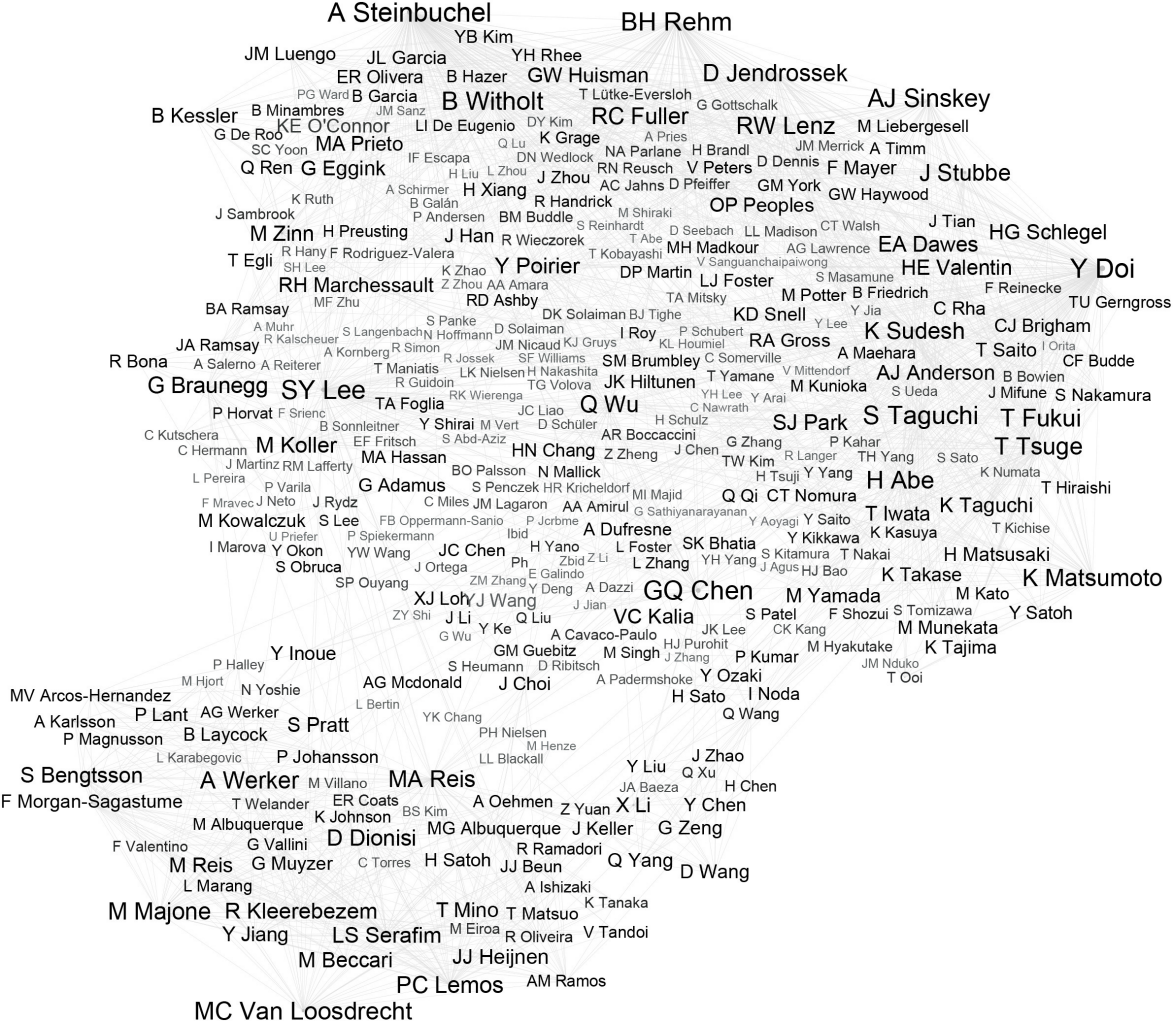
The network of authors of the most frequently quoted in analyzed journals. From each publication, analyzed pairs were identified consisting of: (1) author of the paper and (2) author of referenced paper, represented by the lines in the graphic. All identical pairs in whole corpus were summed up. The greater the sum, the stronger the relation is, which is visualized by the increased font size in the iconographic. The network of relations allows to discover groups of cooperating scientists. These relations are probably based on common university, field of study, etc.

The corpus of the research can be presented as a network of authors of publications and authors indicated by them in the references. This means that not only the most frequently quoted authors but also the relations between them can be discovered. The source of the relations, for example, includes similarity of covered topics, joint research, and long-term cooperation. The network of relations, therefore, helps to understand trends in the literature better. The network was prepared using Gephi (Figure 2). For the sake of clarity, only links with a number of citations >10 were presented. An analysis of all relations would contain over 60,000 authors and over 530,000 relationships. However, it would not significantly impact the list of the most often quoted authors and the most important relations.

### Search for the Most Important Terms

The most important terms are not always those which are the most frequent. In English, the most frequent words are “the” and “of.” In the case of this study, the word “polymer” can be found in every publication. Thus, it does not impact the results. The terms that exist in one publication only are important only for that publication. The most important terms are those which occur in a group of publications. They allow researchers to identify clusters and then trends (Salton and Yang, 1973; Cong et al., 2016). The term frequency–inverted document frequency (TF-IDF) method was designed to solve that problem. It takes into account the frequency of the term but at the same time includes several documents in which that term occurs. The following formula is used:

$$\begin{array}{l} w_{i,j}=tf_{i,j}\cdot \frac{N}{df_{i}} \end{array}$$

where:

*w*_*i,j*_—result for term *i* in document *j*,

*tf*_*i,j*_—number of occurrences of *i* in *j*,

*df*_*i*_—number of documents containing *i*,

*N*—number of documents in corpus (set of documents).

In the result, each publication can be presented as a vector that consists of multiple dimensions. The number of dimensions is equal to the number of terms used in the analysis, usually several thousands. The similarity of the publications as vectors can be assessed using mathematical methods, e.g., cosine similarity (Mihalcea et al., 2006). Some other methods can be used instead of TF-IDF, e.g., Latent Semantic Indexing (LSI) or Latent Dirichlet Allocation (LDA). They can explain the meaning of a text, but the format of results makes their use in further steps of our cluster analysis difficult.

### Discovery of Thematic Groups (Clusters)

The similarity of the vectors can help discover thematic groups of publications. It can be done using partitioning or hierarchical clustering methods. As a result of partitioning, all elements of the corpus has to be assigned to one of the predefined partitions, even if it is not similar to the other elements. The hierarchical clustering includes in clusters only those elements that are similar. Therefore, hierarchical clustering is better when searching for trends. The examples of partitioning methods include k-means, affinity propagation, spectral clustering, and agglomerative clustering. In contrast, the examples of clustering are mean shift (based on k-means), DBSCAN, Optics, and HDBSCAN (McInnes et al., 2017).

We decided to use HDBSCAN (Hierarchical Density-Based Spatial Clustering of Applications with Noise), which is a relatively new method (McInnes et al., 2017). The algorithm takes each publication (vector) and checks at what distance it can find similar ones. Then it compares the results, and the densest areas are detected as clusters. The density and number of elements in the cluster can differ. The researcher has to define the minimum cluster size, which should be identified experimentally. In the case of this study, the minimum cluster size was set to 5.

**Figure 3:**
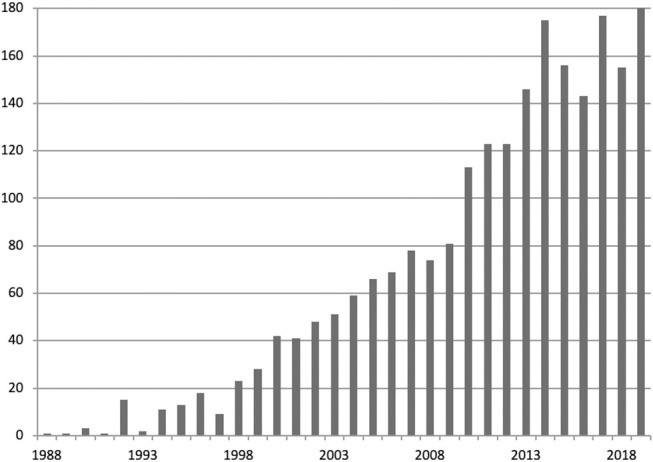
Number of publications in polyhydroxyalkanoate research identified in Web of Science Core Collection for years 1988–2019.

The sample was divided into groups that contained publications published in 5-years overlapping periods starting with 1995–2000 and ending with 2014–2019. Each publication was assigned to all the groups into which it fitted. Cluster analysis was performed in every group separately. Figure 3 presents the number of articles in each year.

Due to the limited length of the publication, it is not possible to describe HDBSCAN algorithm in detail. Full documentation with examples and comparison to other methods can be found on the dedicated website (hdbscan.readthedocs.io).

### Discovery of Trends

Clusters require further analysis to discover trends. Definition of overlapping periods allows automatic detection of trends based on tracking of publications in subsequent corpora. A trend occurs when the average number of articles exceeds a certain expert-determined level per given time. Each step of the analysis leads to the discovery of slightly different clusters in which publication tracking is possible. Thanks to tracking, an evolution of clusters can be observed, and trends can be identified. As a result of the analysis, several types of trends can be discovered:

- long-lasting trends that exist and evolve during the studied period,
- declining trends which end during the studied period,
- emerging trends which begin during the studied period,
- ephemeris trends that begin and end during the studied period.

The final step is trends verification, description, and interpretation, which has to be performed by researchers without the help of algorithms. The researcher has to decide whether trends have been identified correctly. The algorithm usually identifies more trends, as it is very sensitive. They have to be merged by researchers. High sensitivity is intentional, as a lower one could lead to an unjustified merger. Trends can be described based on the most important terms found during the TF-IDF analysis of publications that constitute the trend. The interpretation phase should help to highlight changes within the trends and try to predict their future evolution.

## Trends Unveiled

**Figure 4:**
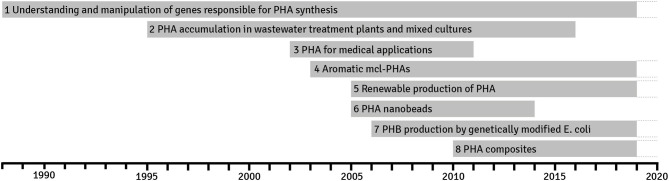
Trends discovered in research on polyhydroxyalkanoates in years 1988–2019 based on the most important terms found during the term frequency–inverted document frequency (TF-IDF) analysis of publications. A trend occurs when the average number of articles exceeds a certain expert-determined level per given time.

The unbiased data mining allowed us to identify eight trends that have been spanning since 1988 (Figure 4). Below, the reader will find their brief description with links to the more specialist reviews and original publications. Some of them are continuing and will last into the future; the others have peaked for several years and are not anymore persistent in the published literature. Nevertheless, it does not mean that research in these areas is not conducted and probably more detailed and narrower studies, which polish the findings over the trend life span, are conducted.

### Understanding and Manipulation of Genes Responsible for Polyhydroxyalkanoate Synthesis

#### Years 1988–2019

This trend is the longest appearing in the research of PHAs over the years. The first works relate to understanding the biosynthesis of PHAs in microorganisms, of which more than 300 have been investigated. Primary findings were described in 1988. Slater et al. (1988) succeeded in the expression of poly-3-hydroxybutyrate (PHB) synthesis genes in *Escherichia coli* by introducing plasmids; at the same time, Schubert et al. (1988) also synthesized PHB in *E. coli* using cosmids. Since then, several biosynthetic pathways have been discovered to date. One distinct was described for short chain length PHAs, the biosynthesis enzymes encoded in the *phaABC* operon, whereas for medium chain length PHAs, the most abundant are: pathways of metabolite shunts through β-oxidation, *de novo* fatty acid synthesis, acyl chain elongation, and others that provide precursors of (*R*)-3-hydroxyacyls from numerous vital biochemical pathways within a given microorganism (see review, Suriyamongkol et al., 2007; Lu et al., 2009). In parallel to the discovery of microbial enzymatic apparatus leading to the synthesis of these polyesters, three key enzymatic groups, directly responsible for PHA synthesis, are also in the spotlight: PHA synthases (see in Stubbe and Tian, 2003; Nomura and Taguchi, 2007), PHA depolymerases (see in Jendrossek, 1998; Ong et al., 2017), and granule associated proteins (see in Dinjaski and Prieto, 2015; Maestro and Sanz, 2017; Gonzalez-Miro et al., 2019). With the development of molecular genetic tools, the constantly discovered genes were being transferred to other model prokaryotes (e.g., *E. coli*, see trend *PHB production by genetically modified Escherichia coli*) and eukaryotes (yeasts, see Breuer et al., 2002, or plants, see Suriyamongkol et al., 2007) in order to increase production yields and thus lower the costs of PHAs. Moreover, directed evolution of enzymes, hybrids generation, and genome manipulations [i.e., through CRISPR/Cas9-based systems (Qin et al., 2018)] not only led to the creation of new robust production strains characterized by good productivities but also allowed for the development of sophisticated polymer bead/enzyme-based platforms (see review Gonzalez-Miro et al., 2019, and also trend *PHA nanobeads*).

### Polyhydroxyalkanoate Accumulation in Wastewater Treatment Plants and Mixed Cultures

#### Years 1995–2016

The increasing world population causes the production of large quantities of urban and industrial waste. Recycling waste and wastewater opens up an opportunity for reducing their quantity and the treatment costs (Cavaillé et al., 2013). Activated sludge can be an alternative to pure cultures for PHA production due to many PHA-producing bacteria present in activated sludge (Yang et al., 2013). In the wastewater treatment process, the microorganisms from activated sludge display the ability to transform a biodegradable carbon source into PHA before using them for growth (Qu and Liu, 2009). Industrial-scale PHA production is based mainly on pure-culture systems with refined feedstock and sterile cultivation conditions. Both features result in high-energy consumption, thereby strongly increasing production costs (Valentino et al., 2019).

Consequently, PHA cannot be cost-competitive to conventional petroleum-based plastics. An innovative approach has been proposed to combine PHA production with sludge minimization in municipal wastewater treatment. In the publications that make up this trend, both the impact of changing the approach to wastewater on the natural environment (Valentino et al., 2015) and the life cycle assessment (LCA) analysis of the production process (Morgan-Sagastume et al., 2016a), as well as specific examples of biochemical (Yang et al., 2013; Inoue et al., 2016) and industrial processes (Pittmann and Steinmetz, 2014; Valentino et al., 2019) resulting in biopolymers, are presented. In the publications, an economic analysis of the process on the example of selected European countries is also carried out (Pittmann and Steinmetz, 2017). The first reports of a significant relationship between PHA production and activated sludge were published in 1998. The issue of biodegradable polymer production with the use of sludge was raised in this publication, as well as the laboratory scale process was described along with performance analysis depending on the reaction conditions (Satoh et al., 1998). Later studies (after 2000) focus on parameters such as carbon and nitrogen sources, their mutual quantitative relations in the fermentation reaction, and the impact of these parameters on process efficiency (Khardenavis et al., 2005). Further analysis on the example of PHB production describes the impact of critical factors such as dissolved oxygen, pH, and food to microorganism (F/M) ratio in the batch reactor on the reaction efficiency (Qu and Liu, 2009). Researchers also carried out physiochemical properties analysis of the polymers PHB and P(HB-co-HV) to identify production conditions that directly affect final product mechanical properties (Wallen and Rohwedder, 1974; Patel et al., 2009). In works mentioned in this trend, the potential of mixed colony nitrogen-fixing bacteria cultures for producing biodegradable polymers with mechanical and chemical properties similar to those originating from pure culture counterparts is presented. In the latest reports (2019), a description of operating pilot plant designed for the production of biopolymer from wastewater can be found (Valentino et al., 2019).

As a summary, one can indicate that a more economical and much cheaper way of producing biopolymers does not necessarily mean a deterioration of their properties, which further indicates the high desirability of projects involving the study and optimization of the production process of bioplastics from sludge (Patel et al., 2009).

### Polyhydroxyalkanoate for Medical Applications

#### Years 2002–2011

PHAs attract the attention of researchers and medical doctors mainly due to their biocompatibility. This feature allows for the creation of medical devices and implants that are completely safe for use in mammals, including humans. PHAs such as P(3HB) and its breakdown products, 3-hydroxy acids, have been found in many organisms—from bacteria to higher mammals. Furthermore, (*R*)-3-hydroxybutyric acid is a natural blood component at concentrations between 0.3 and 1.3 mM. This means that PHAs are excellent biocompatible materials and can, therefore, be successfully used for the construction of cell scaffolds, biodegradable sutures, wound dressings, and drug delivery systems (Zinn et al., 2001; Chen and Wu, 2005). *In vitro* and *in vivo* studies have shown that among the different PHAs, P(3HB-co-3HHx) retains the chondrocyte phenotype, due to the support of chondrocyte-specific extracellular matrix (ECM), type II collagen, and the promotion of sulfated glycosaminoglycan (sGAG) production (Deng et al., 2003). The unquestionable advantage of a polymer such as P(3HB) is its piezoelectric properties, which are similar to those of bones (van der Walle et al., 2001). Therefore, PHAs have been tested for the use of bone loss engineering, and the results showed no chronic inflammation even after 1 year of use (Porter et al., 2013). It was also noted that PHA polymers are widely used in the construction of nerve tissue regeneration devices, dressing materials, cardiovascular patches, venous–arterial valves, orthopedic pins, adhesion barriers, tendon repair materials, bone marrow scaffolds, cardiovascular stents, and tissue engineering (Chen and Wu, 2005; Valappil et al., 2006; Hazer, 2010; Shrivastav et al., 2013). Due to the wide interest of the biomedical industry in PHA polymers, the chemocompatibility of these polymers was also evaluated by incubating P(3HB) and P(3HB-co-3HV) films with mammalian blood during which it was shown that P(3HB) and P(3HB-co-3HV) in contact with blood did not cause negative reactions (Shrivastav et al., 2013).

The first material made of PHA to receive a positive opinion from the US Food and Drug Administration (FDA) was TephaFLEX®, which is an absorbable surgical floss made of P(4HB). There are other commercially available products made of PHA, i.e., GalaFLEX®, which is made of P(4HB) and is designed for reconstructive surgery, plastic surgery, and soft tissue reinforcement, as well as MonoMax Suture for soft tissue regeneration, BioFiber for tendon repair, and Phasix Mesh for hernia regeneration (Williams et al., 2016). Due to their biocompatibility and the hydrophobicity of PHA, they can be used as drug delivery systems in the form of microcapsules, microspheres, and nanoparticles (Shrivastav et al., 2013). In the course of studies on the release of antibiotics such as gentamicin and sulperazone from sticks constructed with P(3HB-co-3HV), continuous release of these drugs over 2 weeks was observed, and it was proved that the content of longer PHA monomers is conducive to prolonging drug release time (Gursel et al., 2002). It has also been shown that lower crystallinity of PHA results in more controlled release of drugs to surrounding tissues. An example was a mixture of P(3HHx-co-3HO) with tamulosin, which was characterized by better penetration of the active substance into the skin, compared to a mixture of the drug with a short-chain, highly crystalline P(3HB) (Wang et al., 2003b). For further details, the reader is encouraged to see the following reviews: Chen et al. (2017); Lizarraga et al. (2018); Elmowafy et al. (2019), and Grigore et al. (2019).

### Processing and Modifications of Polyhydroxyalkanoate Polymers

#### Years 2003–2019

A natural way to add desirable functionalities to PHA polymers is through monomer alterations. More than 150 monomers have been identified (Steinbüchel et al., 1995) that offer a broad range of physicochemical properties (Dinjaski and Prieto, 2015). Among these, aliphatic monomers are in the spotlight. However, these monomers offer only certain properties to scl-PHAs and mcl-PHAs. The other most researched group of PHA monomers is that of aromatic chemistry. We have identified this subtrend in our *in silico* analysis (2003–2009), where most of the publications regarding aromatic monomers were produced. However, the first report that disclosed a biosynthesized PHA bearing an aromatic group as a side chain was presented by Fritzsche et al. (1990) in 1990. In later reports, one can find various aromatic constituents being introduced into PHA chains through biosynthesis achieved mainly with use of *Pseudomonas* strains (Ishii-Hyakutake et al., 2018), recombinant *E. coli* and *Ralstonia eutropha* (Mizuno et al., 2018; Yang et al., 2018). Aromatic PHAs show different mechanical characteristics depending on the type of aromatic monomers incorporated in their structure (Ishii-Hyakutake et al., 2018). In numerous literature reports, the aromatic PHAs have been studied widely. Properties such as degradability (Olivera et al., 2001), surface structure (Takagi et al., 2004), solubility (Mizuno et al., 2017), and thermal behavior (Antoun et al., 1991) have been assessed, and their dependency on the polymer's structure was identified (Ishii-Hyakutake et al., 2018).

Another way to improve the properties of PHAs is to create composites with other materials by physical blending with nanoparticles and nanofillers, produce PHA-based multiphase materials, and perform chemical modifications (Table 2). Modifying PHA is justified with concern regarding features such as their durability, shelf lifetime, replacement, and maintenance costs (Visakh and Roy, 2015). Majority of works on improving and modifying PHAs aims to increase their competitiveness concerning traditional plastics both in terms of physicochemical properties and production costs (Pandey et al., 2005). Incorporating nanoparticles into PHA polymer expands the potential application area thanks to the significant enhancement of materials properties. The most commonly used nanofillers are silylated kaolinite (Zhang et al., 2009), carbon nanotubes (Misra et al., 2010), bioactive glass (Misra et al., 2007), nanoclay (Bordes et al., 2009), cellulose nanocrystals (Yu et al., 2011), calcium phosphates (Cichoń et al., 2019), and modified hydroxides (Dagnon et al., 2009).

**Table 2:** Most common methods used for mechanical modification of polyhydroxyalkanoate (PHA) polymers.

**Modification method**	**PHA polymer used**	**Expected result**	**Potential application**	**References**
Hyaluronan coating	P(HB-co-HHx)	Hydrophilicity ↑	Biomaterial design	Wang et al., 2003a
Blending	P(HB-co-HHx)	Biocompatibility ↑	Biomaterial design	Zhao et al., 2002
Crosslinking (γ-irradiation)	mcl-PHA	Biodegradability ↓ Young Modulus ↑	Biomedical devices design	Ashby et al., 1998
PEGMA grafting (UV irradiation)	mcl-PHA (PHO)	Blood protein adsorption ↓ Biocompatibility ↑	Blood contacting devices	Kim et al., 2005
OH- ion implantation	PHB	Bioactivity ↑ Wettability ↑	Cell culture scaffolds	Hou et al., 2006
O_2_ plasma treatment	P(HB-co-HV)	Hydrophilicity ↑ Surface roughness ↓ Cell proliferation ↑	Retinal pigment epithelium cell culture scaffolds	Tezcaner et al., 2003
Electrospinning	lcl-PHA, mcl-PHA	Elasticity ↑ Wettability ↑	Tissue engineering	Kwon et al., 2007

**Figure 5:**
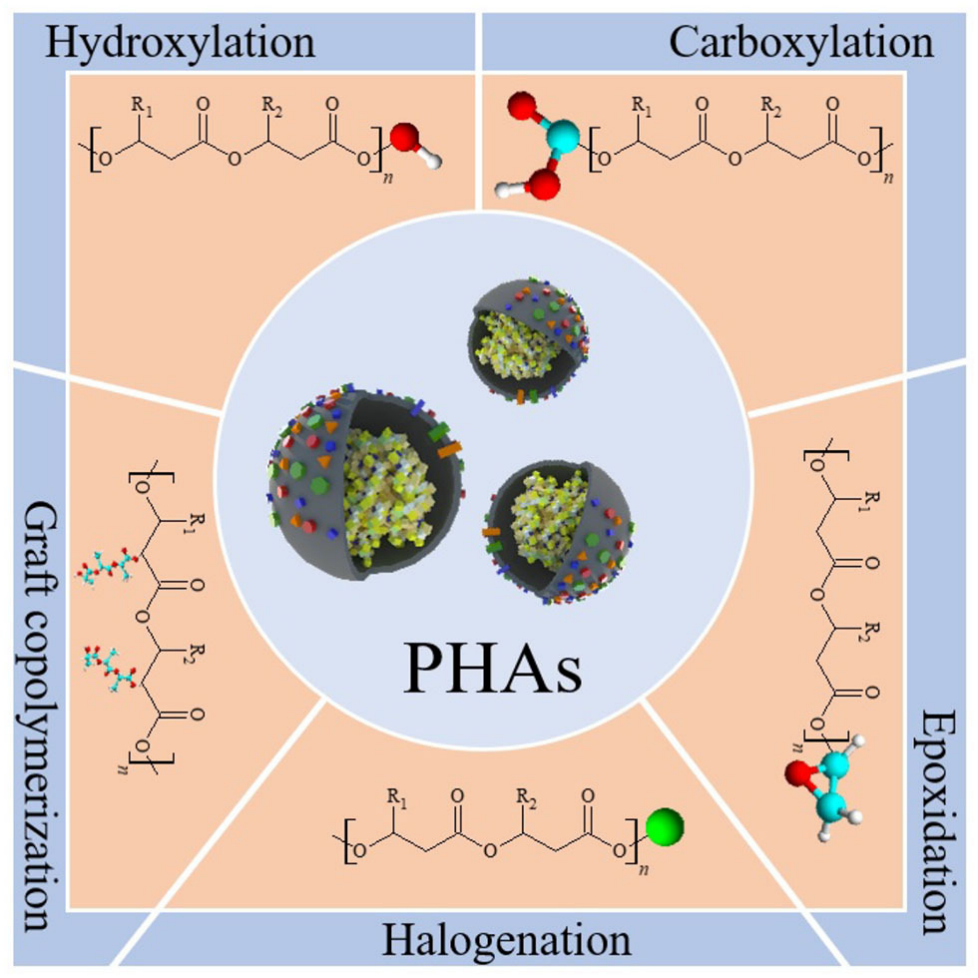
Modification of a polyhydroxyalkanoate (PHA) structure through selected chemical reactions. Carboxylation by addition of carboxylic functional group to the polymeric macromer which can serve as functional binding sites for bioactive moieties. Hydroxylation resulting in shortened hydroxy-terminated PHA chains important in block copolymerization reactions. Epoxidation is useful in diverse reactions such as cross-linking, copolymerization, and bioactive moieties attachment thanks to the high reactivity of the epoxide group. Halogenation which relies on the addition of halogen atoms such as chlorine, bromine, or fluorine to the olefinic bonds of unsaturated PHA, being an excellent method in diversifying functions and applications of the polymer. Graft copolymerization results in the formation of a modified segmented copolymer. Chemical-, radiation-, or plasma discharge-induced reaction evokes improvement of selected polymer properties, i.e., increased wettability or mechanical strength.

To give PHAs new and unique properties, several multiphase materials have been developed, mainly by mixing PHB or P(HB-co-HV) with other products such as plasticizers, fillers, or other polymers (Visakh and Roy, 2015). The structure of a PHA can be modified to obtain a polymer with desired properties for niche applications by chemical processes such as carboxylation, hydroxylation, epoxidation, chlorination, and grafting reaction (Figure 5) (Hazer and Steinbüchel, 2007; Bassas-Galià et al., 2015; Antwi Peprah et al., 2018).

A large group of PHA-based materials was obtained from hydroxylated PHAs and have been reviewed in numerous scientific reports (Cheng et al., 2013). An important factor applicable in PHA modification is high reactivity of epoxide group which can participate in numerous reactions like cross-linking (Visakh and Roy, 2015). PHA modification through epoxidation was reported in several cases, i.e., by Bear et al. (1997) and Park et al. (1999). In a different study, Lee et al. (1999) presented a cross-linking reaction of epoxidized PHA using hexamethylene diamine (HMDA). Another excellent method in diversifying the polymer functions and applications is its modification through halogenation (Arkin et al., 2000; Arkin and Hazer, 2002). In their study, Arkin and Hazer modified the PHA-Cl into ammonium salts, thiosulfate moieties, and phenyl derivatives (Arkin et al., 2000). A process of P(HB-co-HHx) direct fluorination was described by Samsuddin et al. (2013) PHA chemical modification by sulfanyl halogenation and its potential application in electrophotographic imaging were also reported and have been patented in 2008 (Mihara et al., 2006).

Enzymatic PHA modification is a mild, specific, and environment-friendly method widely discussed in the literature. The biopolymer can be modified on an enzymatic route by enzymatic degradation synthesis or by using the degradation products itself (Sato et al., 2008; Gumel et al., 2013; Kwiecień et al., 2015). Enzymatic catalyzed surface erosion of PHA can significantly enhance the surface roughness, thereby cell adhesion and proliferation. The raw PHA surface lacks a bioactive ligand to couple with molecules in targeting devices or biosensors (Visakh and Roy, 2015). Therefore, surface erosion and roughening are essential to immobilize bioactive molecules such as insulin, collagen, or fibronectin to expand the polymer's biomedical applications. The demand for customized biodegradable PHAs with unique features and the difficulty in their conventional production by biosynthesis have shifted the current interest in polymer modification and functionalization *via* chemical, physical, and enzymatic processes (Visakh and Roy, 2015). By precise control over properties such as wettability, elasticity, biocompatibility, biodegradability, or Young's modulus value, one can get a set of smart materials with properties so diverse that they will not only be able to replace traditional polymers but can also contribute to developing new technologies and therapeutic techniques whose use was limited by the lack of material with desired functionality.

### Renewable Production of Polyhydroxyalkanoate

#### Years 2005–2019

PHAs are a promising alternative to petroleum-based polymers; however, their production is still expensive and at industrial scale often competes with the food chain supply. Therefore, a trend emerged in the research of PHAs in 2005 that lasts up to date where research is focused on the search for sustainable production of PHAs from renewable resources. Two parallel platforms are constantly bettered, one that searches for natural wild-type strains capable of efficient conversion of substrates to PHAs and the other that seeks to modify microorganisms genetically. The process optimizations are achieved through not only genetic manipulations but also *via* different fermentation process developments. These include novel strategies for batch, fed-batch, and continuous fermentation optimization with the aid of mathematical modeling of single or mixed microbial cultures. Different strategies for limiting inorganic nutrients are also applied. However, the most crucial issue addressed by research is the application of sustainable carbon sources for the production of renewable PHAs. Therefore, many processes are developed to be integrated into biorefineries as they envisage the use of primary or secondary substrates of existing biotechnologies.

The primary carbon sources for the production of PHA can be various types of vegetable oils such as soybean, coconut, palm, rapeseed, or rubber, which are characterized by low production costs (Kahar et al., 2004; Lee et al., 2008; Kynadi and Suchithra, 2017). The fatty acids also are seen as primary resources used for PHA production—they are obtained from vegetable oils. They include, among others, palmitic, stearic, oleic, linoleic, as well as α-linolenic, caproic, caprylic, and myristic acids (Kynadi and Suchithra, 2017). Employing these substrates for PHA production, one can obtain polymers such as P(3HB-co-3HV), P(3HB-co-3HHx), P(3HB), or mcl-PHAs (Kahar et al., 2004; Ng et al., 2011).

The substrates of the second generation include food, industrial, forestry, and timber waste as well as municipal sewage. Various food waste and food by-products can be used as raw materials for the biotechnological production of PHAs. Mixed food waste is characterized by high complexity and variety of physical properties, particle size, and composition, so pretreatment of such raw material is a necessary step (Nikodinovic-Runic et al., 2013). The second stage of the process of PHA production from food bio-wastes is to obtain a sufficient amount of bacterial biomass capable of production and maximum accumulation of PHA inside the cells. One of the approaches is the use of pure bacterial cultures, their multiplication to an appropriate amount of active biomass on media with easily assimilated carbon source, and their acclimatization before the third stage of the process (PHA synthesis) (Cardozo et al., 2016; Schmidt et al., 2016). The last step usually takes place in a different reactor than the second stage. Still, with a strategy using pure bacterial culture, the approach of PHA synthesis in the same reactor as biomass multiplication is also used, where the polymer synthesis is driven by inorganic nutrient limitation (Rodriguez-Perez et al., 2018).

Wastes with more unified characteristics are also used in the production of PHA. These are, for example, industrial wastes mainly from food production and processing plants (sugar factories, distilleries, slaughterhouses, dairies and cold stores, and oil mills). Others are plant waste from crops, which are generated by agriculture and represent a considerable biomass resource. Agricultural residues include stems, leaves, and seed pods, while process waste also includes peelings, husks, seeds, marc, roots, bark, and sawdust. All these wastes are rich in cellulose, starch, and other carbohydrates. Examples of processes for PHA production based on bacterial monocultures from these substrates are collected in Table 3. The substrates either are fermented directly (glycerol, fatty acids) or require appropriate preprocessing (hydrolysate production). For details, please see the following reviews: Nikodinovic-Runic et al. (2013); Koller (2017); Koller et al. (2017); Kourmentza et al. (2017); Blunt et al. (2018), and Favaro et al. (2019).

**Table 3:** Examples of secondary raw materials and processes for polyhydroxyalkanoate (PHA) production.

**PHA production strain**	**Resource**	**Resources pretreatment**	**References**
*Haloferax mediterranei*	Whey	Acidic hydrolysis	Pais et al., 2015
Recombinant *E. coli* and *R. eutropha*	Rice bran	Acidic hydrolysis	Oh et al., 2015
*Bacillus megaterium*	Molasses	Enrichment of molasses with mono and disaccharides	Gouda et al., 2001
*Burkholderia sacchari* IPT 101	Bagasse	Filtration through activated carbon, acidic hydrolysis	Silva et al., 2004
*Pseudomonas aeruginosa* 42A2 NCIB40045	Used cooking oil	–	Fernandez et al., 2005
*Cupriavidus necator* IPT 029 or *Bacillus megaterium* IPT 429	Crude glycerol	–	Ribeiroa et al., 2016
*Haloferax mediterranei*	Starch	Hydrolysis	Chen et al., 2006
*Burkholderia cepacia* ATCC 17759	Wood hydrolysate	Hydrolysis	Pan et al., 2012
*Haloferax mediterranei*	Rice bran and starch hydrolysate	Enzymatic hydrolysis	Huang et al., 2006

In recent years, there was an increasing number of reports published that provide LCA for PHA-based products and process (Pietrini et al., 2007; Cristóbal et al., 2015; Morgan-Sagastume et al., 2016b; Nitkiewicz et al., 2020). Mainly these studies point toward the implementation of waste streams (resources) for the production of biopolymers. Further, they highlight also that the selection of materials and, eventually, the final product also plays a crucial role in both environmental and economic outcomes of the whole process. Furthermore, the LCA analyses conclude that PHA polymers can find their right place in a biorefinery context, where their production can be carried out in parallel to other biotechnologies, while sourcing substrates from coexisting blocks' waste streams. In this way, there would be not only complemented with zero-waste policies but also upcycling of the generated wastes within biorefineries to high-value products.

### Polyhydroxyalkanoate Nanobeads

#### Years 2005–2014

Inspired by the creation of PHA granules *in vivo* by microorganisms, a trend emerged that encompassed technologies based on spherical nanobeads. First, reports reached for the science behind simple nano/microbeads creation where these vehicles were prepared by emulsification. Thanks to PHA being hydrophobic in nature, it was proposed to mix by physical blending bioactive compounds to produce nano/micro-preparations for antimicrobial and anticancer treatments. Moreover, technologies have been adapted from polymer chemistry in order to functionalize PHA polymers by graft and/or block polymerization with other polymers [e.g., with polyethylene glycol (PEG), poly(ethylene imine) (PEI), or Jeffamine] or covalently bind drugs to the polymeric chains and further create nano/micro-carriers. Recently, hollow or highly open porous microspheres based on PHA biocompatible polymers were constructed as cell carriers for targeted therapies. Tapping into molecular biology and material design, a platform was created that enabled the creation of functionalized nano/microbeads *in vitro* and *in vivo* by enzymatic apparatus of microorganisms. Several works were performed where a genetically modified PHA synthase was used to prepare functionalized PHA beads in a test tube from PHA monomers that either carried a bioactive drug inside a granule or the granule itself was decorated with engineered protein. Finally, a very sophisticated approach was proposed, where microorganism itself through genetic manipulations became able to synthetize a native PHA granule decorated with proteins of interest through their conjugation to granule-associated proteins (phasins or PHA synthetases), enabling the use of such PHA beads in the creation of vaccines, new methods in imaging, bioseparations, or protein purification. More details on the above technologies can be found in the following reviews: Li and Loh (2017); Michalak et al. (2017); Wei et al. (2018), and Gonzalez-Miro et al. (2019).

### Poly-(*R*)-3-Hydroxybutyrate Production by Genetically Modified *Escherichia coli*

#### Years 2006–2019

PHAs are polymerized by synthases, which use various hydroxyacyl-CoAs as substrates. The PHB production from *E. coli* has been attracting attention, even though *E. coli* does not produce PHB naturally. However, its recombinant engineering allowed for the synthesis of this biopolymer. In publications making up this trend, various *E. coli* strain modifications are examined to improve and optimize both the fermentation process and the final product itself. In the majority of the studies, stress is put on the modification of bacteria for processing cheap and readily available carbon sources such as substrates deriving from biomass (Nikel et al., 2006; Park et al., 2012; Yang et al., 2014; Favaro et al., 2019).

The first reports of *E. coli* targeted recombination date back to 1999 when the first attempt of modification of metabolic network on PHB biosynthesis in recombinant *E. coli* was performed. The genes responsible for PHB biosynthesis in *R. eutropha* were cloned in *E. coli* and subsequently sequenced and characterized (Shi et al., 1999). An important source of carbon and nitrogen for bioplastic producing bacteria can be waste and agricultural by-products such as whey and steep corn liquor. It has been presented that PHB can be efficiently produced by the recombinant strain grown aerobically on a laboratory scale bioreactor on a medium supplemented with the agroindustrial by-products (Nikel et al., 2006). A recombinant *E. coli* strain containing the PHA biosynthetic genes from an *Azotobacter* species was specially prepared for producing PHB from milk whey (Nikel et al., 2006). Genetic engineering of *E. coli* can be used for PHA accumulation at low cost and high productivity. The biosynthesis of PHAs containing 2HB monomer from glucose by metabolically engineered *E. coli* strains has been reported (Park et al., 2012). In 2013, the whole process of PHB production from bacterial strain was modeled and simulated. A detailed analysis of each nutrient was performed using response surface methodology (Heshiki, 2013). Modification of metabolic pathways can be used not only to improve the production of known biopolymers but also to program microorganisms to perform a variety of functions such as producing new materials with unique properties (Rahman, 2014). *E. coli* was also metabolically engineered to synthesize copolymers such as [P(3HB-co-3HV)] from glucose (Yang et al., 2014). Most of the studies in this trend focus on the applications of modified *E. coli* bacteria to reduce the cost of PHA production and the possibility of using cheap carbon and nitrogen sources in the fermentation process (Mezzolla et al., 2017; Favaro et al., 2019).

### Polyhydroxyalkanoate Composites—Organic and Nonorganic Blends

#### Years 2010–2019

High price, limited processing capabilities, and poor mechanical properties of PHAs restrain their use for everyday products such as food packaging, disposable cutlery, or device enclosures (Cunha et al., 2016). Additionally, tailored biopolymer composites can play an important role in medical applications such as drug delivery systems, wound healing products, or surgical implant devices (Hufenus et al., 2012). Blending is a simple and effective approach for obtaining new polymeric materials with improved properties, and the drawbacks of the primary components can be eliminated. Typically, the mechanical properties of polymer blends can be easily tuned by varying the compositions of the blend and preparation conditions. Additionally, blends with biodegradable additives can enhance some of the PHA features, such as biodegradability and biocompatibility (Li et al., 2016). In studies included in this trend, several PHA blends were described.

PHA–PLA blends are the most common and the cheapest products for application replacing traditional, petroleum-based plastics (Long, 2009). Predominantly, reports described physical blending of scl-PHAs with PLA polymers which to some extent enhanced the single polymer properties. However, drawbacks such as high melting temperature and brittleness of these blends could be overcome by supplementing them with either mcl-PHAs or other bio-additives (Abdelwahab et al., 2012). This effect can also be achieved by blending Polycaprolactone (PCL) with scl-PHAs, which is an excellent option to improve the mechanical performance of both homopolymers (Visakh and Roy, 2015). Other biopolymers used to create blends with PHAs were cellulose (Zhang et al., 1997), starch (Visakh and Roy, 2015), and chitosan (Ikejima et al., 1999). These polymers have been shown to enhance the PHA properties by lowering the crystallinity of scl-PHAs and enhancing their biodegradability.

Creating blends with PHAs has gained popularity in current world trends. They serve both to reduce the cost of PHA production so that they can compete with petroleum-derived plastics and to improve their physical, chemical, or biological properties to expand the area of potential applications. Blending of PHA with other biodegradable polymers is particularly prevalent when it comes to the creation of novel materials suitable for specific applications.

## Concluding Remarks and Future Perspective

The approach proposed in the study, which was based on the analysis of the full texts of articles, allowed to overcome the limitations of the traditional method based only on a systematic review of literature. The sample selected for analysis was divided into groups containing articles published in 5-years overlapping periods beginning in 1988 and ending in 2019. The analysis allowed to identify eight main areas in PHA research that governed the 31 years of discoveries. It should be noted that the indicated research groups do not exhaust all the conducted studies in the analyzed period. However, they present the research with the highest empowerment in identifiable scientific articles.

The most important outcome of the data-mining process was the identification of these research areas that are still trending in the scientific community and are of high probability to continue. Firstly, scientists try to understand genetics and biochemistry behind PHA synthesis. This leads to the identification of key enzymes responsible for PHA synthesis, thus to the creation of better production strains. Moreover, insight into PHA accumulation from a genetic perspective opens new routes for obtaining multipurpose bioactive granules. Secondly, it is visible from identified trends that a lot of work is being conducted on processing and modifications of PHA, leading to smart composites. These processes and products will lead directly to providing solutions to substitute petrochemical polymers and provide markets with a range of smart materials. Such an approach is also backed up by studies leading to the sustainable production of these biopolymers from renewable and cheap substrates, which directly can reduce their so far high production costs.

It needs to be emphasized that the indicated trends are to serve as a supportive tool for seeking further research directions, which should allow scientists to revise their research areas, improve the research process, and avoid duplication in studies, thereby increasing the efficiency of scientific work. Furthermore, it is vital to remember the given definition of a “trend.” Trends were discovered with restricting criterion of at least 10 papers published in each 5-years period. The criterion was adopted to allow the presentation of the most popular trends. That does not mean that other trends are less important. Moreover, those less popular trends may become leading ones in the future. As cited here, some works, both original or reviews, were published post the trend apogee. They presented either a summary of a specific research area or further narrowed and specialized research continuing from a particular trend. It is also not a foregone conclusion that some of the presented trends will reemerge or new ones will manifest from this broad research on microbial polyesters.

## Data Availability Statement

The raw data supporting the conclusions of this article will be made available by the authors, without undue reservation.

## Author Contributions

SW designed and performed the *in silico* study, grouped literature, arranged, and technically analyzed it. MG and TW searched and provided raw data. SW, MW, and MS wrote methodology and part of introduction and edited the manuscript. MG, TW, and AS performed thematic description of trends, wrote part of introduction, the result, and discussion sections. MW provided funding for *in silico* analysis part. MG gave idea for the study and provided funding.

## Conflict of Interest

The authors declare that the research was conducted in the absence of any commercial or financial relationships that could be construed as a potential conflict of interest.
